# New contributions to the leafhopper genus *Gladkara* from Thailand (Hemiptera, Cicadellidae, Typhlocybinae, Erythroneurini)

**DOI:** 10.3897/zookeys.725.20777

**Published:** 2017-12-29

**Authors:** Yuehua Song, Zizhong Li, Christopher H. Dietrich, Can Li

**Affiliations:** 1 School of Karst Science, Guizhou Normal University/ State Eniineering Technology Institute for karst Desertfication controal, Guiyang, Guizhou 550001, China; 2 Illinois Natural History Survey, Prairie Research Institute, University of Illinois, 1816 S. Oak St., Champaign, IL 61820, USA; 3 Institute of Entomology, Guizhou University, Guiyang, Guizhou 550025, China; 4 Guizhou Provincial Key Laboratory for Rare Animal and Economic Insect of the Mountainous Region, Guiyang University, Guiyang, China

**Keywords:** Auchenorrhyncha, Homoptera, morphology, new species, taxonomy

## Abstract

General characteristics of *Gladkara* Dworakowska and a checklist to all the known species of this genus are provided. A new species *Gladkara klongensis* Song & Dietrich, **sp. n.** from Thailand is added.

## Introduction

The leafhopper genus *Gladkara* was established by Dworakowska (1995) with *G. albida* Dworakowska as its type species. The genus previously contained 13 species, all of which were distributed in the Oriental Region. In this work, one more new species from Thailand is described and illustrated and a checklist of all known species of this genus worldwide is provided.

## Materials and methods

Morphological terminology used in this work follows Dietrich (2005). Habitus photos were taken using a Canon EOS 5D Mark II camera and the Camlift V2.7.0 software. Multiple photographs of each specimen were compressed into final images with Zerene Stacker (64-bit) software. Body length was measured from the apex of crown to the tip of forewings. Abdomens were removed from specimens and cleared in cold 10% KOH solution overnight. The cleared material was rinsed with water and stored in glycerine. An Olympus SZX12 dissecting microscope was used for specimen study and Olympus BX41 and BX53 stereoscopic microscopes were used alternately for drawing of the dissected male genitalia and wings. The holotype of the new species is deposited at the Queen Sirikit Botanical Garden (**QSBG**), Chiang Mai, Thailand and additional specimens examined are deposited at the Illinois Natural History Survey (**INHS**), Prairie Research Institute, University of Illinois at Urbana-Champaign, USA (**UIUC**) and the School of Karst Science (**SKS**), Guizhou Normal University, Guiyang, China.

## Results

### Gladkara

Taxon classificationAnimaliaHemipteraCicadellidae

Dworakowska, 1995

Gladkara Dworakowska, 1995: 4; Dworakowska 2011: 10

#### Type species.

*Gladkara albida* Dworakowska, 1995

#### Diagnosis.

Dorsum beige or white. Color pattern absent or brown. Head narrower than pronotum, crown produced in midline anteriorly. Face convex. Pronotum with large impressions medially. Forewing with AA vein prominent, all known species with dark patch in 3^rd^ apical cell except the type species. Hind wing venation usual for Erythroneurini.

Male 2S abdominal apodemes broad, reaching 3S posterior margin.

#### Male genitalia.

Genital capsule cylindrical, slightly laterally compressed. Pygofer side broad, with caudal margin oval to square, with numerous sparse long fine setae, with or without enlarged macrosetae scattered at ventrolateral angle; dorsal appendage moveably articulated to the lobe, simple or bifurcate far from base, extended to pygofer apex or extended beyond pygofer apex; ventral appendage absent; segment X appendage present. Subgenital plate lamellate, with small latero-basal articulation with style, about 4–5 macrosetae in single or double row, setae of basal group in an oblique or longitudinal row, intergrading into marginal microsetae. Style long and slim, apical part tapering, smooth or adorned with sculpture; preapical lobe small. Connective lamellate, lateral arms long, stem reduced and central lobe desclerotized to various degree. Aedeagus with or without processes, aedeagal shaft tubular; dorsal apodeme with distinct V-shaped ligaments, connected to anal tube and/or pygofer dorsal appendages; preatrium about as long as or shorter than shaft; gonopore apical or subapical on ventral surface.

**Distribution.** India; Brunei; Vietnam and Thailand.

### Gladkara klongensis

Taxon classificationAnimaliaHemipteraCicadellidae

Song & Dietrich sp. n.

http://zoobank.org/EB8889D7-9CAA-4A8B-BAF0-58520703DE72

Figs 1–8 , 9–18

#### Specimens examined.

Holotype: ♂, THAILAND, Surat Thani, Khao Sok NP Klong Morg Unit, 8°53.725'N, 98°39.025'E, 87 m, Malaise trap, 16-23.xii.2008, coll. Pongphan (QSBG). Paratypes: 3♀♀, same data as holotype (INHS, SKS).

#### Diagnosis.

This species has its own salient characteristics as follows: pygofer with a bifurcate dorsal appendage (Fig. 10), segment X appendage twisted medially and hook-like apically (Fig. 11); style extremely elongated, with many cellular sculpturing apically (Fig. 14); aedeagus with long and tubular shaft, and a single short tooth-like process arising from preatrium (Fig. 18).

#### Description.

**Figures 1–8.**
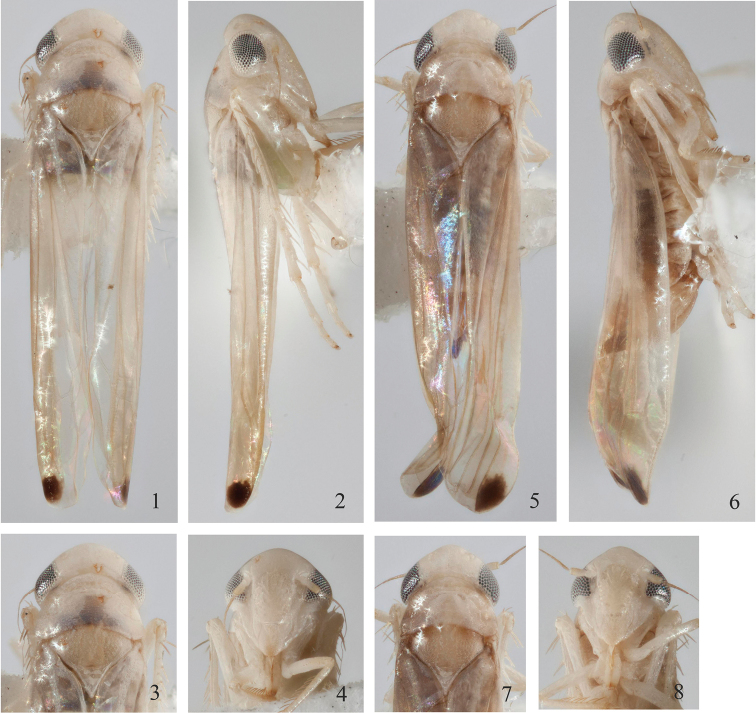
*Gladkara klongensis* Song & Dietrich, sp. n. Male (♂): **1** Habitus, dorsal view **2** Habitus, lateral view **3** Head and thorax, dorsal view **4** Face; Female (♀): **5** Habitus, dorsal view **6** Habitus, lateral view **7** Head and thorax, dorsal view **8** Face.

Ground color pale beige. Eyes grey (Figs 1–3). Face pale beige; apex of frontoclypeus slightly darker in male; coloration extremely pale in female (Figs 4, 8). Pronotum pale anterolaterally, blackish beige medially and posteriorly (Figs 1, 3). Scutellum beige, with prominent scutellar suture (Figs 1, 3). Fore wing beige, with an irregular big dark brown spot at 3^rd^ apical cell (Figs 1, 2).

**Figures 9–18.**
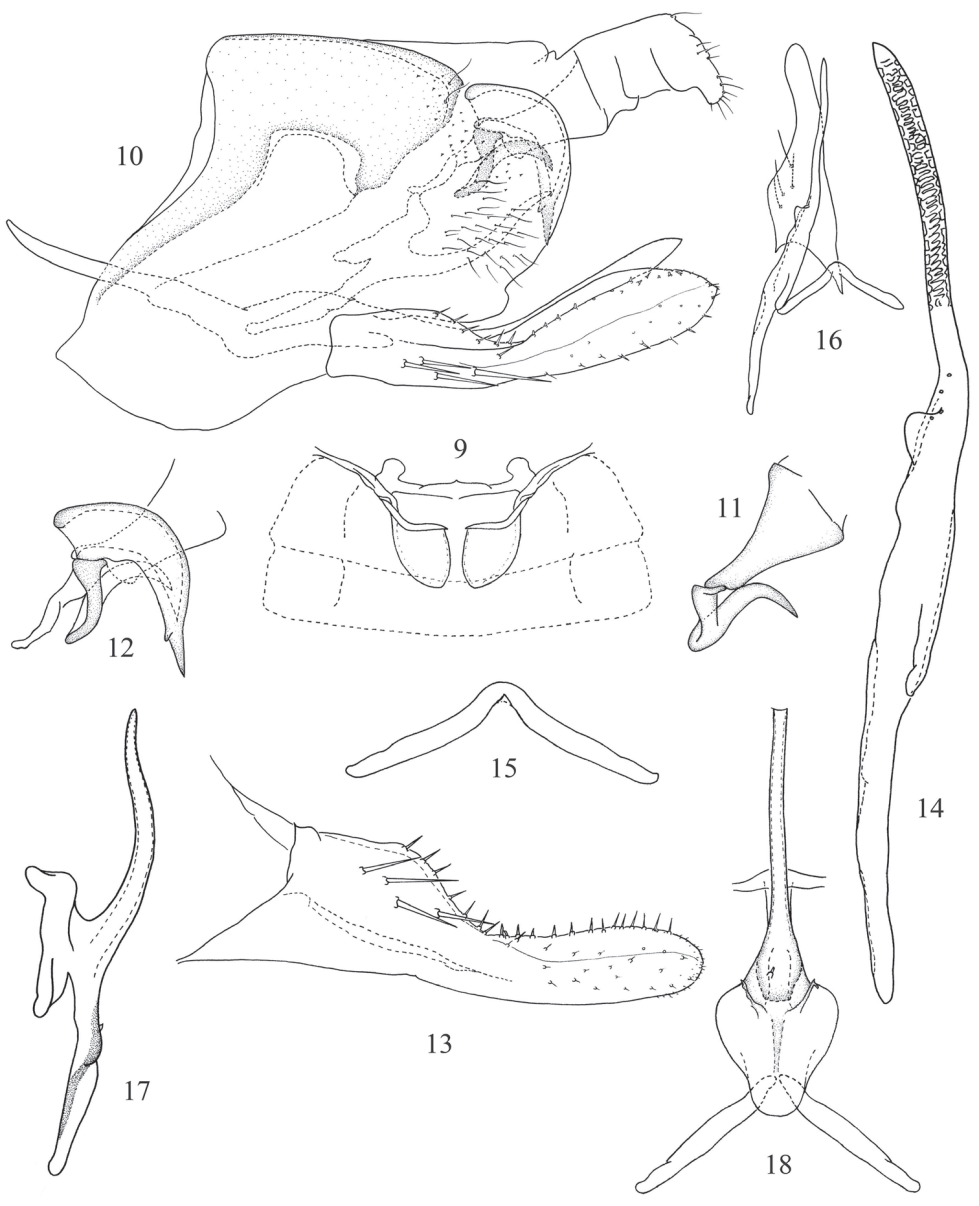
*Gladkara klongensis* Song & Dietrich, sp. n. **9** Abdominal apodemes **10** Genital capsule **11** Segment X appendage **12** Details of connections between segment X, dorsal pygofer appendage and dorsal ligament of aedeagus **13** Subgenital plate **14** Style **15** Connective **16** Subgenital plate, style and connective **17** Aedeagus, lateral view **18** Aedeagus and connective, caudal view.

Male abdominal apodemes broad, extending to the hind margin of third sternite (Fig. 9).

**Male genitalia.** Pygofer with dorsal appendage movably articulated (Fig. 10), segment X appendage elongated, twisted medially, and hook-like apically (Figs 11, 12). Pygofer lobe broadened, with two long fine setae arising from dorso-caudal margin and several long fine setae scattered near caudal margin medially (Fig. 10). Subgenital plate solid, much longer than hind margin of pygofer lobe, with four macrosetae on lateral surface and numerous stout setae along upper margin from sub-base to apex of plate, several microsetae scattered apically (Figs 10, 13). Style extremely elongated, with many cellular sculptures apically; preapical lobe small (Figs 14, 16). Connective V-shaped with lateral arms long and slim, widely divergent; stem short; central lobe absent (Fig. 15). Aedeagus with a single short toothlike process arising from preatrium; preatrium little shorter than length of shaft in lateral view, expanded in ventral view; shaft slender, tubular, slightly tapered distally in lateral view; gonopore apical (Figs 17, 18).

#### Measurements.

Body length, male 3.7 mm; females 3.5–3.6 mm.

#### Remarks.

This species is similar to *G. lisiogon* Dworakowska, 1995 from Brunei (Ulu Temburong) in the form of the aedeagus, but differs in having the dorsal pygofer appendage bifurcate apically (Fig. 12); the single short tooth-like process arising from aedeagal preatrium (Fig. 18); and the aedeagus dorsal apodeme not strongly expanded in caudal view (Fig. 18).

#### Etymology.

The specific name is derived from a part of the type locality name in which all examined species were collected.

##### The species of the genus *Gladkara* worldwide and their distributions

1. *Gladkara albida* Dworakowska, 1995 Distribution: India.

2. *Gladkara bramka* Dworakowska, 1995 Distribution: Brunei.

3. *Gladkara cellularis* Dworakowska, 1995 Distribution: Brunei.

4. *Gladkara hirsuta* Dworakowska, 1995 Distribution: Brunei.

5. *Gladkara interrupta* Dworakowska, 1995 Distribution: Brunei.

6. *Gladkara klara* Dworakowska, 1995 Distribution: Brunei.

7. *Gladkara klongensis* Song & Dietrich, sp. n. Distribution: Thailand.

8. *Gladkara lisiogon* Dworakowska, 1995 Distribution: Brunei.

9. *Gladkara obligata* Dworakowska, 1995 Distribution: Brunei.

10. *Gladkara pulchra* Dworakowska, 1995 Distribution: Brunei.

11. *Gladkara quadrata* Dworakowska, 1995 Distribution: Brunei.

12. *Gladkara sclerosa* Dworakowska, 1995 Distribution: Brunei.

13. *Gladkara vietnamica* Dworakowska, 2011 Distribution: Vietnam.

14. *Gladkara zavijka* Dworakowska, 1995 Distribution: Brunei.

## Supplementary Material

XML Treatment for Gladkara

XML Treatment for Gladkara klongensis
